# Seroprevalence and Associated Risk Factors of Toxocariasis among College Students in Taipei City, Taiwan

**Published:** 2015

**Authors:** Chung-Jung FU, Cheng-Yan KAO, Yueh-Lun LEE, Chien-Wei LIAO, Po-Ching CHEN, Ting-Wu CHUANG, Ying-Chin WANG, Chia-Mei CHOU, Ying-Chie HUANG, Toshio NAITO, Chia-Kwung FAN

**Affiliations:** 1 *Graduate Institute of Biomedical Electronics and Bioinformatics, National Taiwan University, Taipei, Taiwan*; 2 *Dept. of International Medical Affairs, Taipei Medical University-Shuang-Ho Hospital, New Taipei City, Taiwan*; 3 *Dept. of Microbiology and Immunology, *; 4 *Dept. of Molecular Parasitology and Tropical Diseases, School of Medicine, College of Medicine, Taipei Medical University, Taipei, Taiwan*; 5 *Dept. of General Medicine,*; 6 *Dept. of Infection Control Science, Juntendo University School of Medicine, Tokyo, Japan*

**Keywords:** *Toxocara* spp., Western blot, Epilepsy awareness, College students, Taiwan

## Abstract

***Background:*** Infection by *Toxocara* spp. is known to be significantly associated with partial epilepsy. It has become popular for people to raise dogs/cats as pets and consume roasted meat/viscera, and the status of *Toxocara *spp. infection, epilepsy awareness, and associated risk factors among the general population are currently unknown in Taiwan.

***Methods:*** A seroepidemiological investigation among 203 college students (CSs), consisting of 110 males and 93 females with an average age of 21.5 ± 1.2 years, was conducted in 2009 in Taipei City. A Western blot analysis based on excretory-secretory antigens derived from *Toxocara canis* larvae (TcESs) was applied to determine the positivity of serum immunoglobulin G antibodies. A self-administered questionnaire was also given to obtain information about demographic characteristics, epilepsy awareness, and risk factors. A logistic regression model was applied for the statistical analysis using SPSS software.

***Results:*** The overall seropositive rate of *Toxocara* spp. infection was 8.4% (17/203). As to epilepsy awareness, a non-significantly higher seroprevalence was found in CSs who claimed to "know" about epilepsy compared to those who did not know (*P* > 0.05).

***Conclusions: ***It appears that appropriate educational programs are urgently needed to provide correct knowledge related to the prevention and control measures against *Toxocara *spp. infections to avoid potential threats by this parasite to the general population in Taiwan.

## Introduction

Human toxocariasis, which is predominantly caused by *Toxocara* spp. infection, is an important zoonotic parasitosis worldwide. People who live in both developed and developing countries with deficient sanitary, cultural, and social structures are often susceptible to this parasitic infection (1). The literature from surveys worldwide indicates prevalences of *Toxocara *spp. infection in the final host of canids that range 86%~100% in pups and 1%~45% in adult dogs (2-4). Humans, one of the paratenic hosts of *Toxocara *spp., are primarily infected through accidental ingestion of infective eggs of *Toxocara* spp., or by consuming chicken or cow liver or meat containing encapsulated larvae (5-8). Prolonged migration of *Toxocara *spp. larvae in human organs and tissues can result in visceral larva migrans (VLM), and if they reach the eyes or central nervous system (CNS) can cause ocular toxocariasis (OT) or neurotoxocariasis (NT) (1, 9).

Children are usually considered highly susceptible to *Toxocara* infection due to inadequate hygiene knowledge, but adults can also be affected (10). Clinical human toxocariasis cases have not been reported uncommonly in recent years, e.g., endomyocarditis, cataract formation, pulmonary inflammation, hepatosplenomegaly, and meningoencephalitis (11-15). Of note, a large-scale study indicated that *Toxocara *spp. infection poses a significant contributory role in the occurrence of epilepsy in infected patients (16). *Toxocara* larvae cannot grow further in the human host. They are often entrapped by granulomatous tissues in the human body. In addition, it is clinically difficult to detect *Toxocara *spp. larval residue from biopsied tissues. Thus diagnosing *Toxocara* spp. infection primarily relies on a *Toxocara* spp. larval excretory-secretory (TcES) antigen-based enzyme-linked immunosorbent assay (ELISA) (5, 10). Although a sensitivity of 78% and specificity of 92% for the TcES-based ELISA seem quite satisfactory when the titer is set to 1:32 (5, 17), the TcES-based ELISA remains problematic in underdeveloped countries due to common polyparasitism, leading to significant antigenic cross-reactivity (5). Alternatively, issues of helminthic cross-reactivity can be overcome by utilizing fractionated native TcES in a TcES-based Western blot (TcES-WB) analysis because serum immunoglobulin G (IgG) antibodies reactive with low-molecular-weight bands of 24~32 kDa were proven to be specific for *Toxocara *spp. infection (1,5).

People living in urban areas of Taiwan, like Taipei City, like to raise dogs and cats as pets in a trend that has increased with time (18, 19). In addition, the habit of consuming grilled meat/viscera by the general population has become quite popular in Taiwan as grilling imparts a unique flavor and tenderness (20). Nonetheless, meat/viscera often pose potential health risks because of the possible presence of parasites, e.g., encysted *Toxocara* spp. larvae (21). It was assumed that college students (CSs) possess more knowledge related to parasite biology than the general population.

In the present study, we attempted to investigate the seroprevalence of *Toxocara* spp. infection, epilepsy awareness, and associated risk factors among college students from one medical university, which consists of nearly 7000 staff and students, in Taipei City as assessed by detection of sera anti-*Toxocara* IgG antibodies using a TcES-WB analysis and a self-administered questionnaire.

## Materials and Methods

***Study population and subject selection***

This study was conducted among CSs from Taipei Medical University, Taipei City in 2009. After informed consent was obtained from participating students, each one completed a self-administered questionnaire (22). Thereafter, a blood specimen was obtained by venipuncture. Basic demographic data of age, gender, and residence were collected. In total, 203 apparently healthy CSs were enrolled in the study, with a sex ratio of 1.09 (110 males to 93 females) and a mean age of 21.5 ± 1.2 years. Epilepsy awareness and multiple risk factors, e.g., a history of contact with dogs, cats, and soils, the consumption of raw, frozen, or undercooked meat/ viscera, and drinking untreated or unboiled water, were included in the statistical analysis. The mean ages of the genders were similar, and ranged 19~24 years for all participating CSs.

***Ethical approval***

The research protocols were approved by the Institutional Review Board of Taipei Medical University (TMU-No. P970219), and informed consent was obtained from participating CSs.

***Toxocara spp. egg culture***

The culture method used in our previous study (22) was employed. Briefly, adult *Toxocara *spp. were collected from stools of stray dogs treated with mebendazole (Yung-Shin, Taichung, Taiwan). The anterior one-third of the uterus was dissected to harvest eggs by stirring the uterine tissue in a 1% sodium hypochlorite solution and incubating this for 5 min at room temperature; thereafter, the mixture was centrifuged for 5 min at 2000 ×*g*. Subsequently, the pellet was washed twice with distilled water and once with 2% formalin. Eggs were resuspended in 2% formalin and placed in a 250-ml Erlenmeyer flask, to which additional 2% formalin was added to bring the liquid level to approximately 1 cm deep. Finally, the flask was covered with parafilm for incubation at room temperature for 8~9 weeks with gentle weekly agitation. After most of the eggs had fully developed to an infective status, they were stored at 4 °C until being used.

***Preparation of TcES*** ***derived from*** **Toxocara** ***spp. larva*** ***e***

*Toxocara* spp. larvae were hatched according to our previous study (23). Briefly, infective eggs of *Toxocara *spp. were washed with phosphate-buffered saline (PBS), and the pellet was resuspended in 1% sodium hypochlorite and incubated in an atmosphere containing 5% CO_2 _at 37 °C for 30 min. After several washings with PBS containing antibiotics (100 IU/ml penicillin, 250 µg/ml streptomycin, and 25 µg/ml nystatin; Biochrom KG, Berlin, Germany), the pellet was resuspended in RPMI-1640 medium (Sigma-Aldrich Co., St. Louis, MO) containing the same concentrations of antibiotics. Motile larvae were collected from the bottom of the jar containing a modified Baermann apparatus made up of 2 layers of cotton cloth in a steel sieve that had been kept in an atmosphere containing 5% CO_2 _at 37 °C for 12 h. After centrifugation, the pellet containing the larvae was further transferred to new RPMI-1640 medium containing antibiotics in 50-ml tissue culture flasks (BD Biosciences, Franklin Lakes, NJ) with a larval concentration of 10^4^ larvae/ml. Supernatants containing TcES were collected weekly, pooled, and centrifuged to precipitate all debris. The resulting supernatant was sterilized by membrane filtration (through a 0.2-µm pore size) and dialyzed (with a molecular weight cutoff of 6000~8000 kDa) against PBS for 12 h at 4 °C, or until the phenol red disappeared. The protein content of the dialysate was estimated by Bradford's method (1976), and it was then concentrated by lyophilization (Taiwan Green Technology Ltd., Taipei City, Taiwan) and stored at -70 °C until being used.

***WB analysis***

The WB procedure was undertaken according to our previous study (23). Briefly, TcES Ag preparations (9 μg per slab) were separated by 12.5% sodium dodecylsulfate polyacrylamide gel electrophoresis (SDS-PAGE) and transferred to a nitrocellulose membrane (Amersham, Piscataway, NJ) in a semi-blotter (Hoffer, Fremont, CA). Strips were then incubated with sera diluted 1: 64. A Western Lightning^®^ kit (PerkinElmer Life Sciences, Boston, MA) was used to detect the immunoreactions, and positive reactions were ascertained by the presence of low-molecular-weight bands of either 24, 28, 30, or 35 kDa, which were previously confirmed to be specifically correlated to *Toxocara* spp. infection (5).

Statistical analysis

A statistical analysis was performed using the SPSS software system (SPSS, Chicago, IL, USA). Crude odds ratios (ORs) and their 95% confidence intervals (CIs) were estimated by means of a logistic regression analysis, and P values of < 0.05 were considered significant.

## Results

**Fig.1:**
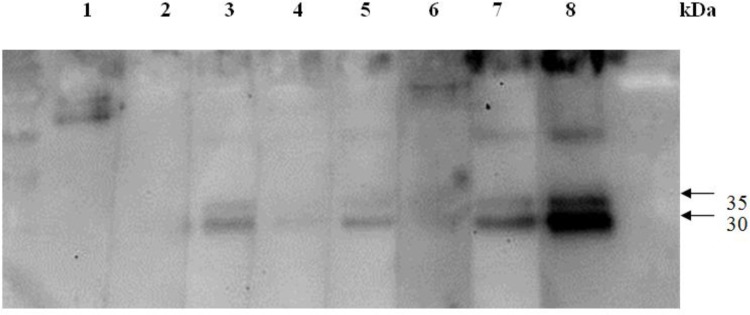
Representative figure indicating results of the immunoblotting analysis of sera from college students from Taipei City whose IgG antibodies were reactive to low-molecular-weight bands at 30 and 35 kDa, which are specifically related to toxocariasis. Lane 1, 2, and 6: negative reactive serum; lane 3-5, and 7: positive reactive serum; lane 8: positive control serum

Among 203 serum specimens tested, IgG antibodies of 17 serum samples were found to be reactive with low molecular weight (24~32 kDa) bands of TcES (Fig.1), resulting in an overall seroprevalence of *Toxocara* spp. infection of 8.4% (17/203) among CSs (Table 1). The logistic regression analysis indicated no gender difference in seroprevalence between males (8.2%, 9/110) and females (8.6%, 8/93) (OR = 1.1, 95% CI = 0.4~2.9, *P* = 0.91) (Table 1). As to epilepsy awareness, a higher seroprevalence was found in CSs who claimed to "know" about epilepsy (8.7%, 14/161) compared to those who did not (7.3%, 3/41), whereas the difference did not reach statistical significance (*P* = 0.78) (Table 1). In the risk factor analysis, consumption of raw or frozen meat, a history of contact with dogs, cats, or soils, drinking unboiled water, and traveling overseas did not exhibit significant differences in seroprevalence (*P* > 0.05) (Table 1).

**Table 1:** Seroprevalence and crude odds ratios (ORs) with 95% confidence interval (CIs) for recognition of partial epilepsy (PE) and various risk factors associated with seropositivity of *Toxocara* spp. (TC) infection among college students in Taipei City

**Variable**	**Group**	**No. tested**	**No. positive (%)**	**OR**	**95% CI**	***P*** ** value**
College students		203	17 (8.4)			
Gender	Male	110	9 (8.2)	reference	-	-
	Female	93	8 (8.6)	1.1	0.4~2.9	0.91
Awareness						
TC associated with PE	No	41	3 (7.3)	reference		
	Yes	161	14 (8.7)	0.8	0.2~3.0	0.78
Risk factors						
Eaten raw meat	No	52	6 (11.5)	reference	-	-
	Yes	144	11 (7.6)	1.6	0.6~4.5	0.39
Eaten frozen meat	No	38	1 (2.6)	reference	-	-
	Yes	141	3 (2.1)	1.2	0.1~12.3	0.85
Contacted soils	No	81	6 (7.4)	reference	-	-
	Yes	115	11 (9.6)	0.8	0.3~2.1	0.60
Contacted dogs	No	115	10 (8.7)	reference	-	-
	Yes	81	7(8.6)	1.0	0.4~2.8	0.99
Contacted cats	No	162	13 (8.0)	reference	-	-
	Yes	34	4 (11.8)	0.7	0.2~2.1	0.48
Drunk unboiled water	No	171	15 (8.8)	reference		
	Yes	25	2 (8.0)	1.1	0.2~5.2	0.90
Traveled overseas	No	166	15(9.0)	reference		
	Yes	29	2 (6.9)	1.3	0.3~6.2	0.71

## Discussion

In their lives, the young general population would rather raise dogs or cats as accompanying animals rather than have a spouse; eventually, when they marry, they still would rather raise pets than have kids; these trends have become more popular in Taiwan particularly under high economic pressures (24). In addition, more people like to taste foreign foods, e.g., Japanese or western style foods, so the trend of consuming grilled meat/viscera including chicken, beef, and pork has also become popular (25). Both trends seem more apparent in urban areas than rural areas in Taiwanese society (24). It thus needs to be considered whether such behavioral changes can cause people to be easily exposed to *Toxocara *spp. infection through these risky routes, which prompted us to investigate the status of *Toxocara *spp. infection among people. CSs would supposedly have better knowledge concerning parasite biology than the general population, and they also have more contact with dogs/cats and eat grilled meat or visceral foods, thus they are a suitable representative target population to understand better the status of *Toxocara* spp. infection and associated risk factors in the general Taiwanese population.

Since *Toxocara* sp. L3 neither grows nor replicates in the human body and it can wander through many internal organs causing human VLM, OT, or NT (1), it is impractical to attempt to detect any ova or larvae from feces. Clinically diagnosing toxocariasis cases predominantly relies on immunological techniques such as TcES-ELISA or TcES-WB analyses. Although the sensitivity and specificity of TcES-ELISA are reportedly reasonable, it is greatly reduced by the problem of antigenic cross-reactivity with various helminthes (5). Despite helminth infections currently being rare in Taiwan owing to better public health knowledge of the general population and improved sanitation systems (26), we still considered using TcES-WB rather than TcES-ELISA to undertake this investigation because of the better specificity of TcES-WB, with reactivity to low-molecular-weight (24~32 kDa) bands proven to be specific for *Toxocara* spp. infection (5).

The present study showed that the overall seroprevalence among CSs was not too much high (8.4%). It is acknowledged that a comparison of seroprevalence data between the present results and other studies is difficult due to different methods employed e.g., ELISA vs. WB, and different cutoff titers used which makes it difficult to evaluate relationships among titer levels, infection, and clinical findings of the disease (5, 27). In studies of adult populations, however, lower rates were recorded, e.g., 1% in Spain (28); while, similar seroprevalences of 5% in South Korea (29) and 7% in Australia (30), but higher rates of 19%, 34%, 81%, and 93% were reported in Lebanon, Bolivia, Nepal, and La Reunion, respectively (31-34). Currently, only one study on the status of *Toxocara* spp. infection among adults who lived in mountainous areas was published in Taiwan, and that study indicated a higher seroprevalence of 30.2% (22). Of note, the serum titer was set to 1: 64 to detect *Toxocara *spp. infection, which reflects those seropositive CSs having possibly acquired an active *Toxocara* spp. infection. In the CDC ELISA, the presence of antibody titers of ≥ 1:32 may be considered indicative of an active *Toxocara* infection (5). Gender did not seem to be an important factor related to *Toxocara *spp. infection among CSs due to a lack of a significant association between gender and frequency of *Toxocara* seropositivity in the present study. However, some studies indicated that males have a greater opportunity to acquire *Toxocara *spp. infection than females as explained by males possibly having more-frequent contact with canine pets and more often consuming undercooked food (32, 34). Moreover, CSs from a medical university would supposedly have greater knowledge related to* Toxocara* spp. biology than the general population, thus making them better able to avoid *Toxocara* spp. infection. The present study showed that the seroprevalence of CSs was indeed not high, and indirectly supported that they seem to be able to avoid *Toxocara* spp. infection because of adequate knowledge.

Nonetheless, CSs who claimed to be aware "*Toxocara* spp. are associated with partial epilepsy"*, *had a relatively higher seroprevalence than those who were not. This result may be due to a misunderstanding or misinterpretation of the phrase “knowing about *Toxocara* spp. being associated with partial epilepsy” by CSs who claimed to "know"; on the other hand, they might have overlooked the importance of preventive measures in avoiding accidentally contracting toxocariasis or their knowledge about *Toxocara *spp. biology might have been incorrect, thus causing them to have more opportunities to be exposed to *Toxocara *spp. infection. It is a serious concern as to whether the general population has a comparatively higher opportunity than CSs to acquire *Toxocara *spp. infection, which warrants further elucidation.

The sample size of our study (203 participants) was not adequate, and risk factors related to toxocariasis were difficult to identify from the present questionnaire investigation. However, contact with soils or cats/dogs is likely risk factors for CSs in contracting toxocariasis, because a risk factor analysis of the data from the 17 seropositive CSs showed that 70.6% (12/17) of them had a history of contact with soils and 61.8% (21/34) with cats/dogs. A variety of studies from different geographical locations have described the presence of embryonated *Toxocara* spp. eggs recovered from the soil or from the fur of dogs suggesting that direct soil or human-dog contact may be important routes of infection for humans (22, 35-37). Since the suitable climatic conditions for *Toxocara* eggs to develop full embryonation are prevalent in Taiwan, it seems likely that male and female CSs have similar frequencies of coming in contact with soils or pet fur, which has been contaminated with *Toxocara* eggs, thus creating increased opportunities to acquire new or repeated *Toxocara* spp. infections. It is a serious concern as to whether such routes of transmission may also play a similar role in the general population, which warrants further investigation.

Undoubtedly, it cannot be ignored that people may acquire *Toxocara *spp. infection through consumption of raw or undercooked infected visceral or muscle tissues containing encapsulated larvae from various paratenic hosts such as chickens, cows, or lambs, although this risk factor did not play a significant role in the transmission of this disease in CSs. However, recently in Japan and Korea, several cases of human toxocariasis associated with the consumption of raw or undercooked meat or liver from chickens, ducks, cows, and rabbits were reported (6-8, 38). It is noteworthy that adults rather than young children were apparently affected by this parasite. This tendency is particularly true for OT. Yoshida et al. (39) described among 38 Japanese OT cases, 34 (89%) were older than 20 years of age, and most of them had risky behaviors such as consuming raw meat or having close contact with contaminated soil.

## Conclusion

It is an urgent issue for the Taiwanese health authorities to consider offering adequate health educational programs including correct information on how to prevent toxocariasis through food- and pet-borne transmission in general as well as specific measures about how to control *Toxocara* spp. infection professionally.
